# Association between Immune-Related Adverse Events and Survival in 319 Stage IV Melanoma Patients Treated with PD-1-Based Immunotherapy: An Approach Based on Clinical Chemistry

**DOI:** 10.3390/cancers13236141

**Published:** 2021-12-06

**Authors:** Lina María Serna-Higuita, Teresa Amaral, Andrea Forschner, Ulrike Leiter, Lukas Flatz, Olivia Seeber, Ioannis Thomas, Claus Garbe, Thomas Kurt Eigentler, Peter Martus

**Affiliations:** 1 Department of Clinical Epidemiology and Applied Biostatistics, Eberhard Karls University of Tuebingen, 72076 Tuebingen, Germany; peter.martus@med.uni-tuebingen.de; 2 Center for Dermatooncology, Department of Dermatology, Eberhard Karls University of Tuebingen, 72076 Tuebingen, Germany; Teresa.amaral@med.uni-tuebingen.de (T.A.); andrea.forschner@med.uni-tuebingen.de (A.F.); Ulrike.Leiter@med.uni-tuebingen.de (U.L.); lukas.flatz@med.uni-tuebingen.de (L.F.); Olivia.Seeber@med.uni-tuebingen.de (O.S.); Ioannis.Thomas@med.uni-tuebingen.de (I.T.); claus.garbe@med.uni-tuebingen.de (C.G.); thomas.eigentler@med.uni-tuebingen.de (T.K.E.); 3 Department of Dermatology, Venereology and Allergology, Charité—Universitätsmedizin Berlin, Corporate Member of Freie Universität Berlin and Humboldt-Universität zu Berlin, 10117 Berlin, Germany

**Keywords:** melanoma, immune-related adverse events, response, survival, immunotherapy, anti-PD-1, anti-CTLA-4

## Abstract

**Simple Summary:**

Nivolumab combined with ipilimumab has improved the prognosis of patients with advanced melanoma. However, this therapy is frequently associated with immune-related adverse events. Published data suggested that objective responses rates appear to be superior in patients who developed immune-related adverse events. The primary aim of this study was to evaluate the association between immune-related adverse events and disease control rate, progressive-free survival, and overall survival in patients with stage IV melanoma treated with first-line PD-1-based immunotherapy. In this manuscript, we show that the presence of immune related side effects is related to better overall response and longer survival in patients with advance stage melanoma treated immuno-therapy, suggesting that immune-related adverse events might be a predictive factor of response in those patients.

**Abstract:**

(1) Background: Immune checkpoint inhibitors have improved the prognosis of patients with advanced melanoma. Published data suggested that the objective response rates appear to be superior in patients who developed immune-related adverse events (irAEs). (2) The primary aim of this cohort study was to evaluate the association between irAEs and disease control rate in patients with stage IV melanoma treated with first-line PD-1-based immunotherapy. (3) Among 319 patients, 53% experienced at least one irAE. A higher percentage of patients with irAEs had disease control compared to those without irAEs (69.8% vs. 49.3%). In multivariate analysis, development of grade 3 and 4 irAEs was significantly associated with a protective effect for the outcome primary resistance (OR: 0.40 95% CI 0.23–0.70, *p* = 0.001). The presence of any grade irAEs was significantly associated with longer OS (irAEs grade 1–2 HRadj: 0.61 95% CI: 0.4–0.93, *p* = 0.02, irAEs grade 3–4 HRadj: 0.55 95% CI 0.31–0.99, *p* = 0.04), but not with PFS (irAEs grade 1–2 HRadj: 1.21 95% CI: 0.91–1.79, *p* = 0.16, irAEs grade 3–4 HRadj: 1.14 95% CI 0.83–2.02, *p* = 0.24). (4) The presence of irAEs with laboratorial expression is positively associated with response and OS, suggesting that irAEs might be a predictive factor in this setting.

## 1. Introduction

Monoclonal antibodies targeting the immune regulatory checkpoint receptors of anti-programmed cell death 1 (PD-1) and anti-cytotoxic T-lymphocyte-associated protein-4 (CTLA-4) have significantly improved the prognosis of patients with advanced melanoma [1,2,3,4]. Currently, three immune checkpoint inhibitors (ICIs) are approved for the treatment of stage IV melanoma: the anti-CTLA4 antibody ipilimumab, and the anti-PD1 antibodies nivolumab and pembrolizumab [1,5,6]. Clinical trials have demonstrated that combined treatment with nivolumab plus ipilimumab and nivolumab monotherapy in patients with advanced melanoma led to better objective response rates (ORR), progression-free (PFS), and overall survival (OS), than treatment with ipilimumab alone [1,7,8,9]. The 5-year OS rate was 52% in the nivolumab plus ipilimumab group, 44% in the nivolumab group, and 26% in the ipilimumab group [7].

Though the introduction of ICI improved the prognosis of patients with metastatic melanoma, this therapy is frequently associated with immune-related adverse events (irAEs) [8,10,11,12]. This can be explained as the ICIs play a role in maintaining immune homeostasis and preventing autoimmunity, therefore their inhibition leads to increased activity of the immune system, resulting in a variety of irAEs that resemble autoimmune diseases in their clinical presentation [6,8,10,11,12,13,14,15]. These irAEs can involve any organ or tissue [16], ranging from mild to life-threatening toxicity [17]. The most commonly irAEs reported are rash, vitiligo, colitis, pneumonitis, hepatitis, thyroiditis, nephritis, and hypophysitis [13,18,19], leading to ICIs discontinuation in approximately 10–20% of patients [16].

A correlation between the diagnosis of severe irAEs and an improvement in PFS and OS in patients receiving ICI has been previously described in diverse tumor entities in the metastatic setting [1,8,13,17,20,21,22]. In an adjuvant setting, and for melanoma patients, Eggermont et al. were also able to demonstrate a correlation between irAEs and an improvement in recurrence-free survival (RFS) [8]. In contrast to reports based on data from clinical studies, this relationship has not been studied as intensively in real-world data [23]. This work describes the association of the occurrence of immune-related adverse events and improved survival in melanoma using real-world data.

## 2. Materials and Methods

### 2.1. Study Design and Data Sources

We conducted a single-center, retrospective cohort study in patients with unresectable stage IV melanoma treated with first-line PD-1-based immunotherapy (pembrolizumab, nivolumab, or nivolumab plus ipilimumab). The German Central Malignant Melanoma Registry (CMMR) was used to initially identify our patients’ collective, i.e., patients diagnosed with stage IV melanoma between January 2015 and December 2018. Additional clinical and laboratory data were retrieved from the patients’ medical records and further documented in the open-source system Epi InfoTM. Consistency analysis was performed with the database, and patients’ medical records from the University Hospital Tuebingen (SAP ISH GUI for Windows) were used to validate and complement the information.

### 2.2. Population

Patients diagnosed with stage IV melanoma (AJCC 8th) [24], who received first-line PD1-based immunotherapy from January 2015 to December 2018, were included (*n* = 353). Patients who received first-line monotherapy with ipilimumab (*n* = 26) and those who had incomplete follow-up data (*n* = 8) were excluded, thus 319 patients were included in this final analysis.

### 2.3. Variables

The following clinical data were collected at baseline: age, sex, histological subtype, tumor localization, BRAF, NRAS and c-kit mutational status, presence and localization of metastasis, date and type of systemic therapy received, best overall response (BOR) to ICI according to Response Evaluation Criteria in Solid Tumors version 1.1 (RECIST 1.1) [25], date of progressive disease (PD), and date of patients’ last contact or death. The BOR to first-line immunotherapy was defined as the best response—intracranial and extracranial —that patients achieved during the time they were treated [26] and was categorized as either complete response (CR), partial response (PR), stable disease (SD), or progressive disease (PD). Patients with CR, PR, and SD were considered to have disease control (DC) [26]. Patients with PD as BOR were considered as having primary resistance to ICI, as they did not respond to first-line ICI. Imaging assessment was performed by a radiologist demonstrating the radiological findings during treatment of each patient in the interdisciplinary tumor board.

Due to the retrospective nature of this study, it was difficult to assess the presence of irAEs for which a clinical evaluation is necessary, for example, cutaneous irAEs, since this depends on the documentation of these adverse events in the patients’ medical records. Therefore, to improve the data quality, we focused on the irAEs for which objective laboratory values were documented.

The laboratorial parameters used to identify irAEs associated with ICI therapy were retrieved from the central laboratory of the University Hospital Tuebingen between January 2015 and March 2019, allowing us a minimum of 3 months of follow-up after therapy start for the last patient included in the analysis. The laboratorial investigations included in this study were hematological (hemoglobin, platelets, leukocytes, neutrophils, lymphocytes, and eosinophils), hepatobiliary (AST, ALT; GGT, direct bilirubin, indirect bilirubin, and alkaline phosphatase), endocrine (TSH, fT3, fT4, and cortisol) and renal (creatinine, glomerular filtration rate, and blood urea nitrogen). All irAEs were graded according to the National Cancer Institute (NCI) Common Terminology Criteria for Adverse Events version 5.0 (CTCAE) [27]. Patients with liver metastases and elevated liver enzymes at the time of immunotherapy start were excluded, as further elevation of transaminases due to ICI therapy could not be clearly differentiated from liver disease progression.

### 2.4. Objectives and Endpoints

The primary endpoint was the DC rate, defined as the percentage of patients with DC, and the secondary endpoints included PFS and OS. PFS was defined as the time between the date of therapy start and date of documented PD according to RECIST 1.1; OS was defined as time from start of therapy and patients’ last contact or death due to any cause. The data cutoff date for the analysis was March 2019.

### 2.5. Detectable Effect

The sample size was fixed by the number of patients from the CMMR (*n* = 319). With this sample size, we had a power of 95% (type I error 0.05 two-sided, chi-square test) to detect group differences in proportions of 0.20 (software PASS 2020). The observed frequency of irAEs (169 with irAEs vs. 150 without irAEs) and the total frequency of patients with DC were used in this calculation.

### 2.6. Statistical Analysis

We first described the patients’ characteristics using the appropriate descriptive statistics according to the type of variables. Qualitative variables were described using absolute and relative frequency. Numerical variables were described as means and standard deviation or medians, and interquartile ranges (IQR) according to the distribution of the data. The normality of the distribution was assessed by investigating skewness and kurtosis as well as QQ graphs, box plots, and histograms. Bivariate analysis was performed by grouping the patients based on the presence or absence of irAEs. Categorical variables were compared using Chi-square tests (or Fisher’s exact test for small data sets). Continuous variables were compared between groups (no irAEs, irAEs grade 1–2, and irAEs grade 3–4) using one-way analysis of variance (ANOVA) for normally distributed data or Kruskal–Wallis test for non-normally distributed data.

To test the associations between irAEs and response, i.e., DC vs. PD, we performed a binary logistic regression model. Confounding variables were selected based on clinical reasoning and statistically significant results in bivariate analyses. Crude (simple regression model) and adjusted (multiple regression model) odds ratios (OR) and 95% confidence intervals (CI) were calculated. The goodness of fit was evaluated using the Hosmer-Lemeshow test (HL).

Cumulative incidence (CI) of irAEs was estimated considering death as a competing risk. The proportional sub-distribution hazard model of Fine and Gray was used to analyze the effect of type of therapy, sex, and age on the incidence of irAEs [28,29]. Censored data (PFS and OS) were analyzed using the Kaplan–Meier method, and the log-rank test was used to test differences of survival distribution by groups (irAEs present vs. not present). In addition, a 3-months landmark survival analysis was performed, excluding patients who were lost to follow-up or died in the first 3 months after starting ICI [30]. Univariate time-dependent Cox proportional regression models were used to evaluate the relationships between the outcomes (PFS and OS) and age, sex, histological subtype, BRAF mutation status, number and localization of metastasis, type of immunotherapy, and irAEs as a time-varying variable. IrAEs were categorized as follows: 0 = no irAEs available, 1 = irAEs grade 1–2, and 2 = irAEs grade 3–4. [8,31]. We used a multivariate time-dependent Cox proportional hazard model to assess differences in OS and PFS risk associated with the presence or absence of irAEs. Variables initially introduced in the multivariate survival analyses were all variables associated with PFS or OS in the univariate analyses with a *p*-value < 0.10 or variables previously identified as risk factors. Interactions between independent covariates were tested in the final models. Hazard ratios (HRs) and 95% CIs were estimated. The proportional hazard assumption was tested for each covariate of the Cox model using the Schoenfeld residual. All reported *p* values were two sided and the significance level was set at ≤0.05. Missing data were assumed to be at random and multiple imputation by chained equations (package “Mice”) was applied to handle missing data [32]. All the analyses were carried out using the statistical program for social sciences IBM SPSS software version 26.0 (IBM, New York, NY, USA) and R software version 3.6.

### 2.7. Ethics Approval

The data were collected as part of routine clinical care in compliance with good clinical practices. The study was approved by the Ethics Committee of the University Hospital Tuebingen (project number 149/2020BO2) and conducted in accordance with the Declaration of Helsinki.

## 3. Results

The final analysis included 319 patients, with a median follow-up of 24 months (95% CI 19–29). Sixty percent of the patients were men (*n* = 192). The mean age of the patients at the time of therapy start was 65.5 (SD 14.4, range 19 to 90) years. The BOR to first-line PD-1-based immunotherapy was PD in 39.8% (127 patients), SD in 19.4% (62 patients), PR in 25.1% (80 patients) and CR in 15.7% (50 patients). Table 1 shows the other baseline characteristics of the study population.

### 3.1. Cumulative Incidence of irAEs

One hundred sixty-nine (53%) patients experienced at least one irAE. Multiple irAEs occurred in the same patient: 1–5 irAEs in 99 patients (31.0%) and more than 5 irAEs in 70 patients (21.9%). The frequency distribution was: hematological (51.1%, 163 patients), renal (28.8%, 92 patients), hepatobiliary (25.4%, 81 patients) and endocrine (24.1%, 77 patients). Type of immunotherapy (PD-1 monotherapy vs. nivolumab plus ipilimumab), age and sex were not associated with the frequency of irAEs (Fine–Gray sub-distribution hazard model *p* = 0.71, *p* = 0.95, *p* = 0.13, respectively). More information can be seen in Figure S1.

The cumulative incidence of grade 1 or higher irAEs in the PD-1 monotherapy group at 1, 3, and 6 months was 23.8%, 39.7%, and 48.9%, and in the nivolumab plus ipilimumab group was 26.9%, 43.2%, and 52.3%, respectively.

### 3.2. Association between irAEs and Response

The DC rate was 60.2% for the whole collective, 69.8% for patients with any grade irAEs, and 49.3% for patients without irAEs (Table 1). The median duration of therapy in DC and PC groups was 7 months (IQR: 4–14) and 2 months (IQR: 1–3), respectively. Patients with DC had a significantly higher irAEs rate compared to those with PD. Considering the type of irAEs, all were strongly associated with response (Table 2).

Table 3 presents the binary logistic regression analysis of potential predictors of DC. After adjusting for confounding factors, the presence of any type of irAEs grade 3–4 was found to be associated with a protective effect for the outcome PD (adjusted OR (ORadj): 0.40, 95% CI 0.23–0.70, *p* = 0.001). This model was adjusted by age, sex, S100 values, number of metastases, and type of immunotherapy. The Hosmer–Lemeshow test showed goodness of fit of the model (HL: χ^2^ = 8.374 *p* = 0.398 df = 8).

### 3.3. Association between irAEs and Progression-Free Survival

**Figure 1:**
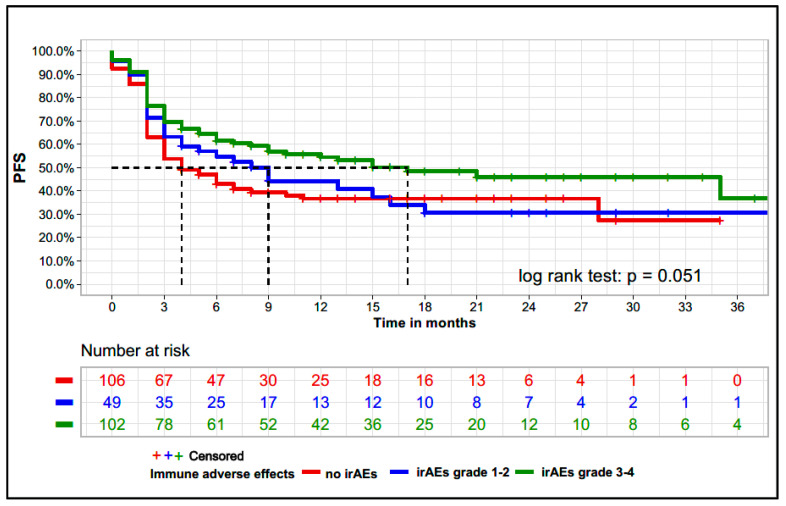
Kaplan–Meier curves for progression-free survival considering the presence of immune-related adverse events and respective CTCAE grade Kaplan–Meier curve for the 3-months landmark survival analysis of irAEs, which excluded patients who were lost to follow-up or died prior to this time of 3 months after starting immunotherapy (*n* = 257). irAEs: immune-related adverse events. Median PFS: not irAEs: 3 months (95% CI, 2–3), irAEs grade 1–2: 6 months (95% CI 3–16) and irAEs grade 3–4: 15 months (95% CI 7-NA).

In our cohort, the 3, 6, and 12 months PFS was 53.0%, 45.0%, and 38.7% respectively with a median PFS (mPFS) of 4 months (95% CI 3–7). We analyzed the association of PFS with irAEs stratified for low grade (CTCAE grade 1–2) and high grade (CTCAE grade 3–4). The mPFS in patients with no irAEs, low-grade irAEs, and high-grade irAEs was 3 months (95% CI, 2–3), 6 months (95% CI 3–16), and 15 months (95% CI 7-NA), respectively. At the 3-months landmark survival analysis, PFS was associated with the presence of irAEs (log-rank test = 0.05) (Figure 1). When PFS was analyzed considering the number of irAEs, patients with >5 irAEs (70 patients) and 1–5 irAEs (99 patients) had a statistically significant longer mPFS compared to patients with no irAEs (150 patients) (mPFS: 10 months [95% CI: 3–17], 9 months [95% CI: 0–18] and 3 months [95% CI: 2–3] *p* < 0.01, respectively).

PFS was also evaluated in the predetermined sub-types of irAEs (hematological, hepatic, endocrine, and renal). In all pre-specified sub-types, patients with grade 3–4 irAEs had better PFS outcomes than those with irAEs grade 1–2 or no irAEs (log-rank test: *p* = 0.02, *p* = 0.005 and *p* = 0.006 for hematological, renal, and endocrine irAEs, respectively), except in the subgroup with hepatic irAEs where patients with grade 3–4 irAEs had worse outcome (*p* = 0.04) (Figure S2). The 12 months PFS rate for patients without hepatic irAEs and those with hepatic irAEs CTCAE grade 1–2 and CTCAE grade 3–4 was 43.7%, 58.6%, and 29.2%, respectively.

**Figure 2:**
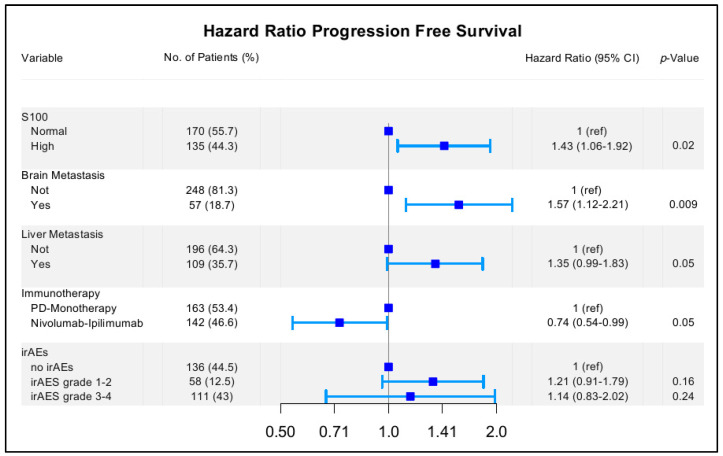
Multivariate analysis, time-dependent Cox regression model (progression-free survival) irAEs: immune-related adverse events.

A time-dependent Cox regression model was used to estimate the association between PFS and irAEs. In the univariate analysis, besides irAEs, S-100 levels and the presence of brain or liver metastasis were also associated with PFS). These variables were further integrated into a multivariate extended Cox regression model. The type of immunotherapy was also included, as this is a clinically relevant variable. The occurrence of irAEs grade 1–2 and 3–4 was not associated with a longer PFS; irAEs grade 1–2: HRadj 1.21 [95% CI: 0.91–1.79] *p* = 0.16 and irAEs grade 3–4 HRadj: 1.14 [95% CI: 0.83–2.02] *p* = 0.24 (Figure 2). On the contrary, immunotherapy with nivolumab plus ipilimumab was independently associated with longer PFS. An additional investigation included the interaction between the presence of irAEs and the type of immunotherapy received (PD-1 monotherapy vs. nivolumab plus ipilimumab), which was not significant (*p* = 0.50). Different tests and graphical strategies were used to check the proportionality assumption of the Cox model. The Schoenfeld residual suggested evidence of proportionality (*p* global = 0.125). In addition, we performed a proportional cause-specific hazards model including irAEs as the time-dependent variable and mortality as the competing risk. The estimated sub-distribution hazard ratio [33] of the variable irAEs was also not significant (irAEs grade 1–2 HR: 1.32 95% CI: 0.94–1.85, *p* = 0.11, irAEs grade 3–4 HR: 1.16 95% CI 0.65–2.03 *p* = 0.62).

### 3.4. Association between irAEs and Overall Survival

**Figure 3:**
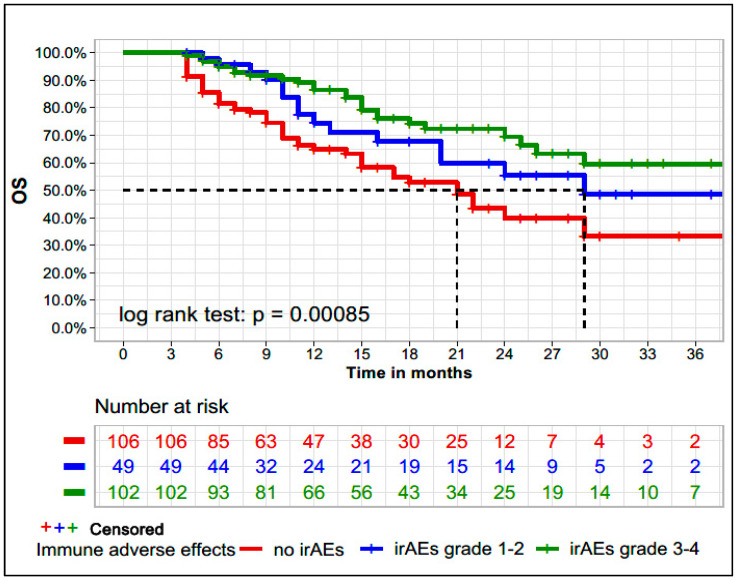
Kaplan-Meier plot for overall survival considering the presence of immune-related adverse events and respective CTCAE grade. Kaplan-Meier curve for the 3-months landmark survival analysis of irAEs, which excluded patients who were lost to follow-up or died prior to this time of 3 months after starting immunotherapy (*n* = 257). irAEs: immune-related adverse events. Median OS: not irAEs: 21 months (95% CI 15-NA), irAEs grade 1–2: 29 months (95% CI 20-NA), and irAEs grade 3–4: not reached (95% CI, 29-not reached).

At the 3-months landmark survival analysis, the median OS (mOS) in patients with no irAEs, irAEs grade 1–2, and irAEs grade 3–4, was 21 months (95% CI 15-NA), 29 months (95% CI 20-NA) and not reached (95% CI, 29-not reached), respectively. OS was associated with the presence of irAEs (log-rank test < 0.001) (Figure 3). The 12-months OS in the groups with no irAE, irAEs grade 1–2, and irAEs grade 3–4 was 64.9%, 74.5%, and 86.5%, respectively. When OS was analyzed according to the number of irAEs developed, patients who developed ≥5 irAEs and 1–5 irAEs had a longer mOS compared to those without irAEs (mOS: NR [95% CI: 29-NR], NR [95% CI: 26-NR], and 21 months [95% CI: 15-NR] *p* < 0.001). Figure S3 displays the association between OS and the predefined sub-groups of irAEs. All sub-types of irAEs were significantly associated with improved OS.

**Figure 4:**
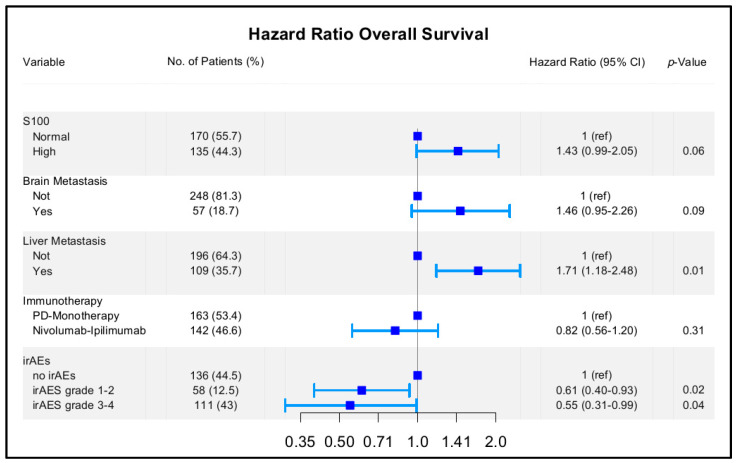
Multivariate analysis, time-dependent Cox regression model (overall survival) irAEs: immune-related adverse events.

Subsequently, we performed OS univariate analysis that included irAEs as a time-dependent variable. The univariate analysis underlined an association between OS and brain or liver metastasis, elevated S-100 values, and the presence of irAEs. The interaction between the type of immunotherapy received (PD-1 monotherapy vs. nivolumab plus ipilimumab) and irAEs were not statistically significant. Multivariable analysis confirmed that irAEs grade 1–2 (HRadj 0.61, 95% CI 0.40–0.93, *p* = 0.02) and irAEs grade 3–4 (HRadj 0.55, 95% CI 0.31–0.99, *p* = 0.04) were significantly associated with increased OS (Figure 4). The Schoenfeld residual suggested evidence of proportionality (*p* global = 0.118).

## 4. Discussion

In the present study, we found that the development of irAEs, as expressed by changes in laboratory values, is significantly associated with disease control in patients with stage IV melanoma treated with PD-1-based immunotherapy indicating that irAEs can be a predictive factor for ICI. The statistically significant association between irAEs and DC was seen in all the pre-defined sub-types of irAEs. The presence of irAEs was also significantly associated with an improved OS and a trend was seen for PFS. Finally, we confirmed the prognostic value of other known factors in stage IV melanoma, such as the presence of elevated S100 levels and the presence of liver and brain metastases.

In our cohort, the rate of irAEs of any grade was 53%, similar to other reports using daily routine data [1,13], but lower than the rates previously reported in clinical trials [34,35,36]. Hematologic and renal immune-related adverse events were reported more frequently in this work than in other comparable studies. The reason for this is probably that we performed a systematic evaluation of all laboratory findings for each patient and found relatively frequent findings of white and red blood cells deviating from the normal value and deviating values for creatinine.

The time to onset of irAEs described in other publications varies between 2 and 16 weeks [18,19,34,37]. In our cohort, the median time to onset of irAEs was 12 weeks (95% CI 12–20), which is longer than previously reported, probably because we did not consider the irAEs that have an earlier onset, such as cutaneous and gastro-intestinal irAEs, and included those with later onset as endocrine and renal irAEs [19,38]. The time of onset of irAEs, however, does not seem to be associated with response, as publications involving different tumor entities, including in melanoma, show conflicting results [21].

The association between irAEs and response to ICI has been concordantly described in melanoma, renal cell carcinoma, and non-small cell lung cancer patients [1,34,39,40,41,42,43]. In a pooled analysis of four trials including 576 patients with advanced melanoma treated with nivolumab, the presence of irAEs was significantly associated with ORR [34]. The ORR in patients with any irAEs was higher compared to those patients with no irAEs (ORR 48.6% vs. 17.8%; 95%CI: 42.3–54.9 and 13.7–22.4). In our cohort, the DC rate in patients with any grade irAEs was 69.8% and 49.3% in those without irAEs. The difference might be related to the sub-type of irAEs reported by Weber et al. and in our cohort, and to the type of treatment received. Weber et al. reported the ORR for patients treated with nivolumab monotherapy, 54% of which had received prior ipilimumab therapy, while in our cohort we only included patients receiving first-line immunotherapy, 45.5% of which received nivolumab plus ipilimumab [34]. The question arises, however, as to whether patients with disease progression had even spent enough time on treatment to develop immune-mediated adverse events. Of the 127 patients with progressive disease, 94 had already died during the observation period; the median survival time of this collective was 7 months. The median progression occurred after 2 months and the median duration of treatment was also 2 months. It is, therefore, possible that this short duration of treatment contributed to fewer immune-mediated adverse events being observed.

The benefit in terms of PFS for patients with irAEs seems to be quite consistent in non-small cell lung cancer, and gastro-intestinal malignancies [41,43,44,45]. As for melanoma, in the pooled analysis reported by Weber et al., there seems to be no benefit in terms of PFS for patients with irAEs [34]. In our analysis, a trend in terms of PFS benefit was seen when comparing patients without irAEs and with grade 1–2 or 3–4 irAEs (*p* = 0.051), but this trend was not confirmed in the multivariate time-dependent Cox analysis. However, Indini et al. reported that the presence of irAEs was the only factor independently associated with improved PFS [1]. The differences in terms of populations included, the sub-type of irAEs reported, the fact that we analyzed the irAEs in two groups (grade 1–2 and grade 3–4) instead of all together, and the different systemic therapies might justify these differences. In addition, PFS was very short for patients with tumor progression especially in those patients with primary resistance, the median PFS was only 2 months. For the total group of patients with onset of tumor progression, the median PFS is 4 months. This short period of time may not be enough to reveal the effects of an immune response.

Contrary to the PFS benefit, the benefit in terms of OS for patients with irAEs seems to be more homogenous, aligned with our current report, where the mOS in the groups with no irAE, irAEs grade 1–2, and grade 3–4 was 21 months, 29 months and not reached, respectively (*p* < 0.001). The presence of irAEs was also independently associated with OS in the multivariate time-dependent Cox regression analysis. Indini et al. also reported a benefit in OS, particularly for patients with vitiligo, compared to those without irAEs (median OS 9.7 months for no irAEs vs. 21.9 months for other irAEs vs. not reached for patients with vitiligo) [1]. In another publication that included data from 148 patients with melanoma treated with nivolumab, a statistically significant OS difference was noted among patients with irAE compared to those without irAEs (*p* < 0.001) [13]. Finally, in the adjuvant setting, particularly in high-risk stage III melanoma, a statistically significant association between irAEs and improved recurrence-free survival and OS was also reported [8].

In our cohort, the association between irAEs and improved OS was present in all predefined categories of irAEs, except in patients with hepatic irAEs. In this subgroup, we saw that grade 1–2 hepatic irAEs but not grade 3–4 irAEs were associated with favorable outcomes. When analyzing the proportion of patients with irAEs grouped by presence or absence of liver metastases, 42.6% of patients with liver metastases had hepatic irAEs compared to 29.9% of patients without liver metastases. For the survival analysis of patients with hepatic irAE, we excluded patients with liver metastases and elevated liver enzymes at baseline. Nevertheless, we cannot completely exclude that the worse outcome seen is due to the presence of unrecognized liver micrometastases and/or later hepatic progressive disease, which can translate into liver enzymes elevation.

Immune modulation resulting from ICIs can alter normal self-tolerance, which clinically translates into irAEs. Despite intense research on the topic, the exact mechanisms by which irAEs are triggered are still not clear [46,47]. Studies suggest that irAEs could be caused by antigens that are common to both tumor and affected organ, leading to a cytotoxic effect on normal cells. Treatment with ICIs increases T-cell activation and proliferation leading to increased production of proinflammatory cytokines triggering a nonspecific activation of the immune response, with non-specific inflammation and auto-immunity [37,48,49]. In addition, anti-PD-1 therapy may also affect humoral immunity, leading to increased levels of pre-existing autoantibodies [50,51]. The combination of these mechanisms would lead to hyperactivation of the immune system, translating into a higher rate of irAEs but also into a better tumor response. The challenge here is to uncouple tumor response and toxicity. Available data are scarce, and it is still unclear if this is at all possible [8]. In our cohort, premature treatment discontinuations were rare (~5%) and we generally observed stable remission in these patients.

Strengths and Limitations: Our study has several strengths. First, the reliability of the data used and the severity grade attributed to each irAEs were not dependent on clinical documentation as it is, for example, for cutaneous, rheumatological, gastro-intestinal, or lung toxicities. Here, we used an established classification for irAEs (CTCAE) which allows for future comparisons with other analyses. Second, the study includes many patients with an adequate median follow-up time, which allowed a precise estimation of the association between irAEs and the outcomes DC, PFS, and OS, as well as subgroup analyses. Third, we used adequate statistical methods to avoid bias, and we adjusted the analyses for possible confounders.

Regarding limitations of this study, it is important to point that this is a retrospective study, thus bias in patients’ selection can be present. Moreover, due to its retrospective nature and the strategy used for irAE identification, not all irAEs were included, and this could have affected our analysis.

## 5. Conclusions

The presence of irAEs was positively and significantly associated with DC and OS. This observation was stable in all the Cox regression models performed. Our data show that the presence of irAEs may predict DC in patients with advanced melanoma receiving ICI. However, to adequately compare and investigate the predictive effect of irAEs across tumor entities and irAEs sub-types, a standardized collection of irAEs is necessary.

## Figures and Tables

**Table 1:** Baseline characteristics of the patients considering the diagnosis of immune-related adverse events during the study (*n* = 319).

Characteristics	*N*	Total	No irAEs (*n* = 150)	irAEs Grade 1–2 (*n* = 58)	irAEs Grade 3–4 (*n* = 111)	*p*-Value
Age at therapy start mean (±SD)	319	65.5 (±14.4)	65.9 (±14.7)	64.9 (±14.4)	65.3 (±14.0)	0.85 ^Anova^
Sex;	319					
Female *n* (%)		127 (39.8)	65 (43.3)	21 (36.2)	41 (36.9)	0.48 ^Chi^
Male *n* (%)		192 (60.2)	85 (56.7)	37 (63.8)	70 (63.1)	
Tumor localization;	319					
Head and neck *n* (%)		54 (16.9)	22 (14.7)	10 (17.2)	22 (19.8)	0.93 ^Fisher^
Trunk *n* (%)		73 (22.9)	35 (23.3)	15 (24.1)	23 (21.6)	
Extremity *n* (%)		109 (34.2)	51 (34.0)	22 (36.2)	36 (33.3)	
Others *n* (%)		15 (4.7)	9 (6.0)	2 (3.4)	4 (3.6)	
Unknown *n* (%)		68 (21.3)	33 (22.0)	11 (18.9)	24 (21.6)	
Histological subtype;	319					
SSM *n* (%)		77 (24.1)	38 (25.3)	11 (18.9)	28 (25.2)	0.0005 ^Fisher^
NM *n* (%)		73) (22.9)	30 (20.0)	14 (24.1)	29 (26.1)	
ALM *n* (%)		30 (9.4)	12 (8.0)	9 (13.8)	9 (9.0)	
LMM *n* (%)		13 (4.1)	4 (2.7)	5 (8.8)	4 (3.6)	
Uveal or ciliar body *n* (%)		13 (4.1)	0 (0)	9 (15.5)	4 (3.6)	
Others * *n* (%)		40 (12.5)	21 (14.0)	7 (12.1)	12 (10.8)	
Unknown *n* (%)		73 (22.9)	45 (30.0)	5 (6.9)	23 (21.6)	
BRAF status;	319					
WT *n* (%)		197 (61.8)	89 (59.3)	36 (62.1)	72 (64.9)	0.51 ^Chi^
BRAFV600 *n* (%)		93 (29.2)	49 (32.7)	13 (22.4)	31 (27.9)	
Unknown *n* (%)		29 (9.1)	12 (8.0)	9 (15.5)	8 (7.2)	
Kit mutation;	319					
WT *n* (%)		158 (49.5)	64 (42.7)	28 (48.3)	66 (59.5)	0.84 ^Fisher^
Yes *n* (%)		15 (4.7)	7 (4.7)	3 (5.1)	5 (4.5)	
Unknown *n* (%)		146 (45.8)	79 (52.7)	27 (46.6)	40 (36.0)	
LDH baseline;	299					
Normal *n* (%)		204 (68.2)	85 (65.5)	41 (70.7)	78 (70.3)	0.65 ^Chi^
Elevated *n* (%)		95 (31.8)	45 (34.6)	17 (29.3)	33 (29.7)	
S100 baseline;	305					
Normal *n* (%)		170 (55.7)	70 (51.5)	33 (56.9)	67 (60.4)	0.37 ^Chi^
Elevated *n* (%)		135 (44.3)	66 (48.5)	25 (43.1)	44 (39.6)	
Number of organs with metastases;	319					
1–3 *n* (%)		285 (89.3)	130 (86.7)	52 (89.7)	103 (92.8)	0.28 ^Chi^
>3 *n* (%)		34 (10.7)	20 (13.3)	6 (10.3)	8 (7.2)	
Patients with brain metastases *n* (%)	319	61 (19.1)	41 (27.3)	11 (19.0)	9 (8.1)	<0.001 ^Chi^
Patients with liver metastases *n* (%)	319	115 (36.1)	50 (33.3)	24 (41.4)	41 (36.9)	0.54 ^Chi^
First-line immunotherapy	319					
PD-1 monotherapy *n* (%)		174 (54.6)	82 (54.7)	31 (53.4)	61 (54.9)	0.98 ^Chi^
Nivolumab plus ipilimumab *n* (%)		145 (45.5)	68 (45.3)	27 (46.6)	50 (45.1)	
Best overall response	319					
Complete response *n* (%)		50 (15.7)	18 (12.0)	7 (12.1)	25 (22.5)	<0.001 ^LL^
Partial response *n* (%)		80 (25.1)	22 (14.7)	19 (32.8)	39 (35.1)	
Stable disease *n* (%)		62 (19.4)	34 (22.7)	12 (20.7)	16 (14.4)	
Progressive disease *n* (%)		127 (39.8)	76 (50.7)	20 (34.5)	31 (27.9)	

WT: wild-type; PD-1 programmed cell death protein 1. irAEs: immune-related adverse events, Anova: one-way analysis of variance, Chi: Chi square-test, LL: linear-by-linear association test, Fisher: Fisher test. * Others: desmoplastic, polipoid, amelanotic melanoma. SSM: Superficial spreading melanoma, NM: Nodular melanoma, ALM: Acral lentiginous melanoma, LMM: Lentigo maligna melanoma.

**Table 2:** Response considering the presence and type of immune-related adverse events.

Type of Immune-Related Adverse Event	Disease Control (*n* = 192)	Progressive Disease (*n* = 127)	*p*
irAEs *n* (%)	118 (61.5)	51 (40.2)	<0.001
Hematological irAEs *n* (%)	117 (60.9)	46 (36.2)	<0.001
Hepatic irAEs *n* (%)	62 (32.3)	19 (14.9)	0.001
Renal irAEs *n* (%)	72 (37.5)	20 (15.7)	<0.001
Endocrine irAEs *n* (%)	60 (31.3)	17 (13.4)	<0.001

Patients with complete response (CR), partial response (PR), and stable disease (SD) as best overall response were considered to have disease control (DC), and patients with progressive disease (PD) were considered to have primary resistance, irAEs: immune-related adverse events.

**Table 3:** Binary logistic regression analysis of protective factors in relation to the outcome progressive disease (*n* = 319).

Variable	Univariate Analysis			Multivariate Analysis		
	OR	95% CI	*p*-Value	OR	95% CI	*p*-Value
Age > 65 years	0.90	0.57–1.41	0.63			
Sex, male	0.63	0.40–0.99	0.05	0.74	0.45–1.23	0.24
S100 high	2.82	1.75–4.53	<0.001	2.51	1.53–4.15	<0.001
Brain metastases	1.16	0.66–2.03	0.62			
Liver metastases	1.42	0.89–2.26	0.14			
More than 3 metastases	1.82	0.89–3.71	0.10	1.59	0.71–3.60	0.26
Immunotherapy first-line						
Nivolumab	1			1		
Nivolumab/ipilimumab	0.74	0.47–1.16	0.19	0.63	0.38–1.04	0.07
Grade of irAEs						
No irAEs	1			1		
Grade 1–2	0.51	0.27–0.96		0.54	0.28–1.04	0.07
Grade 3–4	0.38	0.22–0.64		0.40	0.23–0.70	0.001

irAEs: immune-related adverse events.

## Data Availability

The data presented in this study are available on request from the corresponding author. The data are not publicly available due to data protection.

## Supplementary Materials

The following are available online at https://www.mdpi.com/article/10.3390/cancers13236141/s1, Figure S1: Number of immune-related adverse events by time-period and cumulative incidence of immune-related adverse events, Figure S2: Kaplan–Meier curves for progression-free survival by type of immune-related adverse events, Figure S3: Kaplan–Meier curves for overall survival by type of immune-related adverse events.
