# Supplementary material for: Association between Immune-Related Adverse Events and Survival in 319 Stage IV Melanoma Patients Treated with PD-1-Based Immunotherapy: An Approach Based on Clinical Chemistry
Source: Cancers (Basel). 2021 Dec 6;13(23):6141. doi: 10.3390/cancers13236141 (PMC8657404; doi:10.3390/cancers13236141)
Supplement: Supplementary file 1 [file cancers-13-06141-s001.zip › cancers-1463849-supplementary.pdf]

# Association between Immune-Related Adverse Events and Survival in 319 Stage IV Melanoma Patients Treated with PD-1-Based Immunotherapy: An Approach Based on Clinical Chemistry

**Lina María Serna-Higuita, Teresa Amaral, Andrea Forschner, Ulrike Leiter, Lukas Flatz, Olivia Seeber, Ioannis Thomas, Claus Garbe, Thomas Kurt Eigentler and Peter Martus**

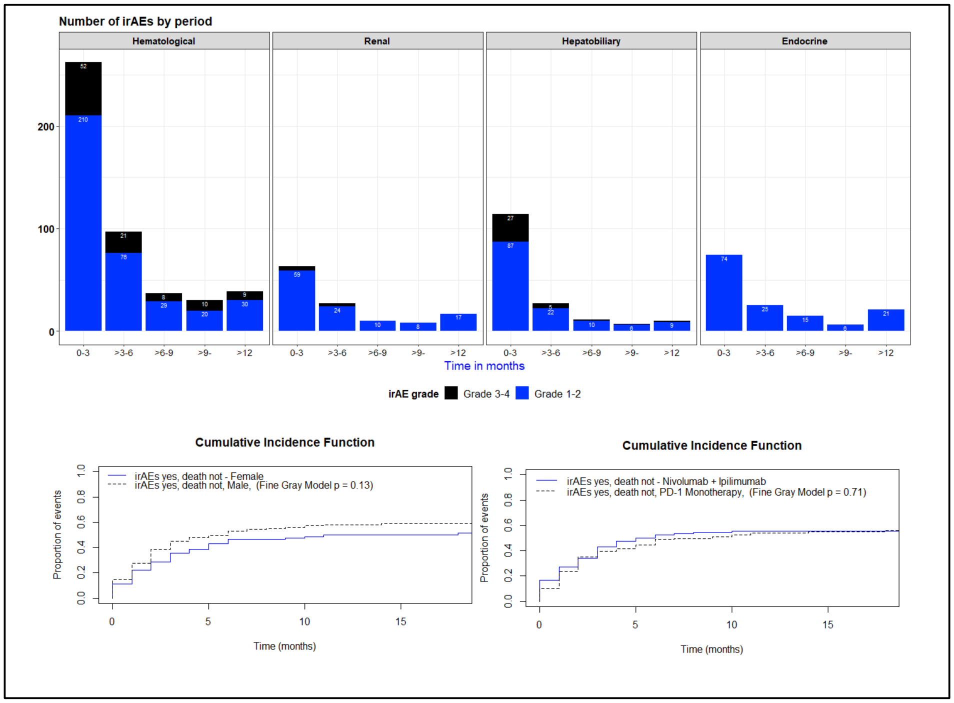


**Figure S1.** Number of immune-related adverse events by time-period and cumulative incidence of immune-related adverse events. *n* = number of patients at therapy by period. Cumulative incidences adjusted for competing risks. Note: 1 patient can have more than one side effect per period.

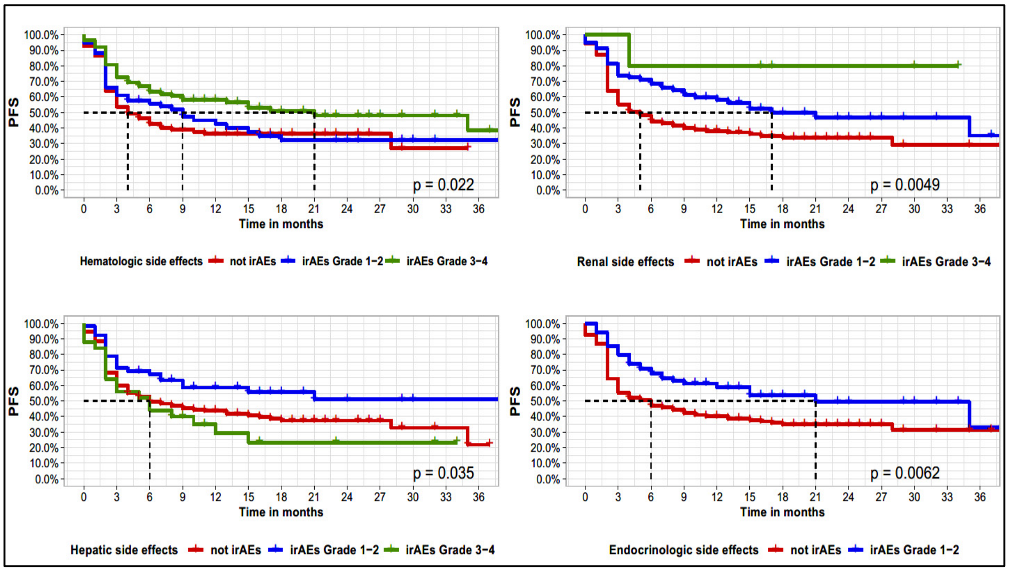

**Figure S2.** Kaplan–Meier curves for progression-free survival by type of immune-related adverse events, excluding patients who were lost to follow-up or died in the first 3 months after starting immunotherapy ($n$ = 257) (landmark survival analysis). irAEs: immune-related adverse events, $p$: Log-rank test $p$-value.

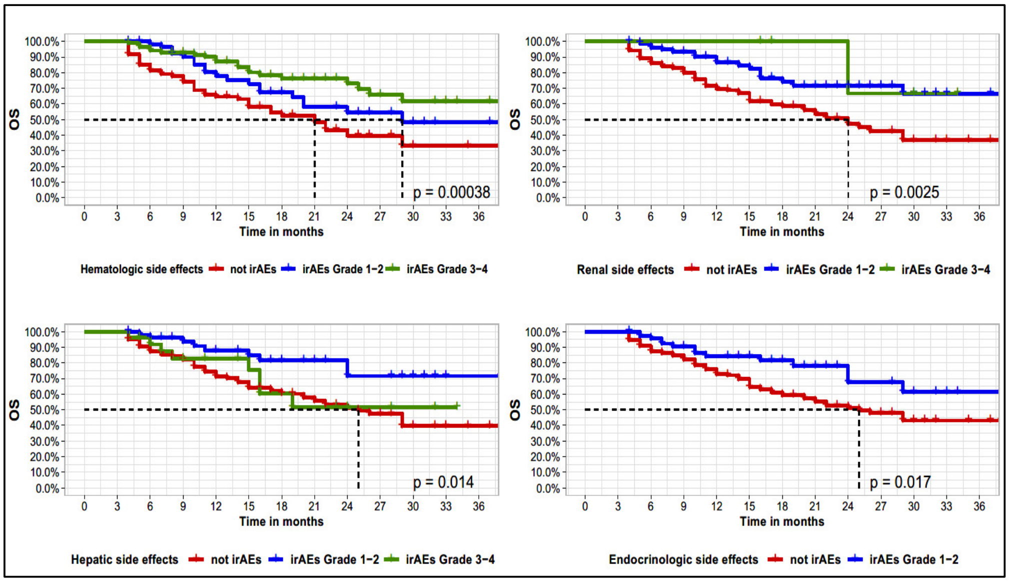


**Figure S3.** Kaplan–Meier curves for overall survival by type of immune-related adverse events, excluding patients who were lost to follow-up or died in the first 3 months after starting immunotherapy ($n$ = 257) (landmark survival analysis). irAEs: immune-related adverse events, $p$: Log-rank test $p$-value.
